# Supplementary material for: Identification of photoperiod-induced specific miRNAs in the adrenal glands of Sunite sheep (Ovis aries)
Source: Front Vet Sci. 2022 Jul 22;9:888207. doi: 10.3389/fvets.2022.888207 (PMC9354845; doi:10.3389/fvets.2022.888207)
Supplement: Supplementary file 1 [file Data_Sheet_1.ZIP › Supplementary file/miRDeep2 file.zip!/miRDeep2 file/Know_Structure/LP42A3/oar-mir-133.pdf]

miRBase precursor : oar-mir-133
Total read count : 497
oar-miR-133 read count : 483
remaining reads : 14

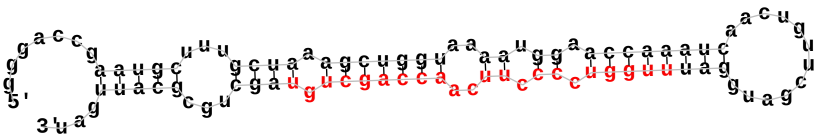

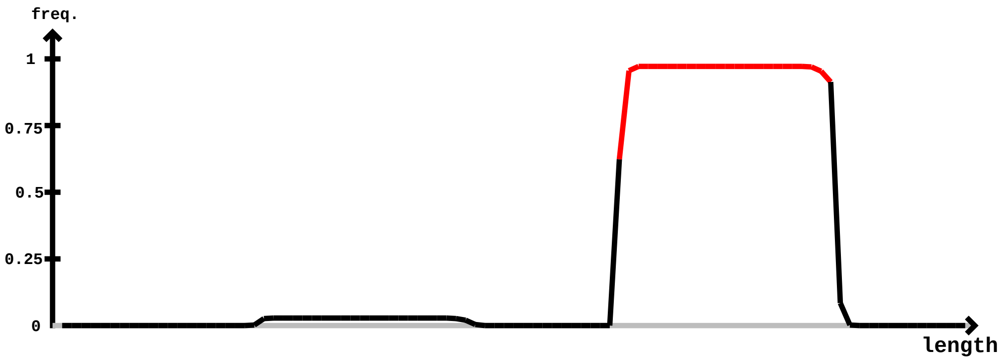


```
                                                  oar-miR-133
5'- gggaccgaaugcuuugcuaaagcugguaaaauggaaccaaaucaacuguucgauggauuugguccccuucaaccagcuguagcugcgcauugau  -3'   exp
    .......(((((...((((.(((((((..((.((.((((((((............)))))))).)).))..))))))).))))...)))))...   reads   mm   sample
    ...................Gagcugguaaaauggaaccaaau....................................................   1       1    seq
    ....................agcugguaaaauggaaccaa......................................................   1       0    seq
    ....................agcugguaaaauggaaccaaa.....................................................   3       0    seq
    ....................agcugguaaaauggaaccaaau....................................................   7       0    seq
    ....................agcugguaaaauggaaccaaauc...................................................   1       0    seq
    .....................gcugguaaaauggaaccaaauc...................................................   1       0    seq
    .........................................................uuugguccccuucaaccagc.................   1       0    seq
    .........................................................uuugguccccuucaaccagcu................   7       0    seq
    .........................................................uuugguccccuucaaccagcuA...............   1       1    seq
    .........................................................uuugguccccuucaaccagcug...............   13      0    seq
    .........................................................uuugguccccuucaaccagcugu..............   273     0    seq
    .........................................................uuugguccccuucaaccagcugua.............   15      0    seq
    ..........................................................uugguccccuucaaccagcu................   1       0    seq
    ..........................................................uugguccccuucaaccagcuA...............   1       1    seq
    ..........................................................uugguccccuucaaccagcug...............   5       0    seq
    ..........................................................uugguccccuucaaccagcugu..............   132     0    seq
    ..........................................................uugguccccuucaaccagcugua.............   25      0    seq
    ..........................................................uugguccccuucaaccagcuguag............   1       0    seq
    ...........................................................ugguccccuucaaccagcugu..............   7       0    seq
    ...........................................................ugguccccuucaaccagcugua.............   1       0    seq
```
